# Sex differences in APP processing in human brains

**DOI:** 10.1002/alz70855_099073

**Published:** 2025-12-23

**Authors:** Sarah Bellaflor, Ahmad Mohammad, Rebecca EK MacPherson

**Affiliations:** ^1^ Brock University, St. Catharines, ON, Canada; ^2^ University of Toronto, Toronto, ON, Canada; ^3^ Brock University, Department of Health Sciences, St. Catharines, ON, Canada; ^4^ Brock University, Centre for Neurosciences, St. Catharines, ON, Canada

## Abstract

**Background:**

Amyloidogenic processing of the transmembrane protein amyloid precursor protein (APP), by BACE1, results in the production of Aβ peptides which are a pathological hallmark of Alzheimer's disease (AD). AD is a biological female dominated disorder, with postmenopausal females accounting for ∼72% of late onset Alzheimer's cases. Due to this sex discrepancy, there has been an increased interest in investigating sex differences in AD pathology. However, studies have not made direct comparisons between APP processing in biological males and females. Animal research has examined sex differences between MALE, SHAM, and OVX models and found cognitive decline in OVX models and higher BACE1 content and activity. The purpose of this study was to examine sex differences in brain markers associated with APP processing in AD and non‐diseased male and female human brains.

**Method:**

Human Brain samples were acquired from the Douglas‐Bell Canada Brain Bank (DBCBB). The collection included hippocampal samples from 9 non‐diseased individuals of both sexes, as well as 19 hippocampal samples from individuals with AD, comprising 9 females and 10 males. Western blots for APP, APP processing markers (sAPPα, sAPPβ, BACE1, ADAM10), and APP processing mediators (proBDNF, matBDNF) were analyzed, along with activity assays for BACE1 and ADAM10.

**Result:**

The AD samples had lower ADAM10 content (*p* =  0.017), and a lower sAPPα: sAPPβ ratio compared to the non‐diseased samples (*p* =  0.008). AD samples also had higher BACE1 activity (*p* =  0.036) and proBDNF content (*p* =  0.022) compared to the non‐diseased samples. The non‐diseased female samples had higher matBDNF in comparison to the AD females (*p* =  0.058). There were no differences in protein content or activity between the sexes.

**Conclusion:**

This study demonstrates differences in APP processing markers between AD and non‐diseased samples. Contrary to our initial hypothesis, no significant sex differences were observed in APP processing markers or enzyme activities between males and females in either the AD or non‐diseased groups. These findings contribute to our understanding of AD pathology and underscore the complexity of sex‐based differences in the disease, suggesting the need for further research to explore the mechanisms underlying AD prevalence.

